# Plasma virome of cattle from forest region revealed diverse small circular ssDNA viral genomes

**DOI:** 10.1186/s12985-018-0923-9

**Published:** 2018-01-15

**Authors:** Hao Wang, Shouxin Li, Asif Mahmood, Shixing Yang, Xiaochun Wang, Quan Shen, Tongling Shan, Xutao Deng, Jingjiao Li, Xiuguo Hua, Li Cui, Eric Delwart, Wen Zhang

**Affiliations:** 1 Department of Microbiology, School of Medicine, Jiangsu University, Zhenjiang, Jiangsu 212013 China; 2 Department of Swine Infectious Disease, Shanghai Veterinary Research Institute, Chinese Academy of Agricultural Sciences, Shanghai, 200241 China; 3 College of Wildlife Resources, Northeast Forestry University, Harbin, Heilongjiang 150040 China; 4 College of Life Science, Shenyang Normal University, Shenyang, Liaoning 110034 China; 5 Blood Systems Research Institute, Department of Laboratory Medicine, University of California San Francisco, San Francisco, CA 94118 USA; 6 School of Agriculture and Biology, Shanghai Jiaotong University, Shanghai, 200240 China

**Keywords:** Cattle blood, Virome, CRESS-DNA virus, Parvovirus

## Abstract

**Background:**

Free-range cattle are common in the Northeast China area, which have close contact with farmers and may carry virus threatening to cattle and farmers.

**Methods:**

Using viral metagenomics we analyzed the virome in plasma samples collected from 80 cattle from the forested region of Northeast China.

**Results:**

The virome of cattle plasma is composed of the viruses belonging to the families including *Parvoviridae, Papillomaviridae, Picobirnaviridae,* and divergent viral genomes showing sequence similarity to circular Rep-encoding single stranded (CRESS) DNA viruses. Five such CRESS-DNA genomes were full characterized, with Rep sequences related to circovirus and gemycircularvirus. Three bovine parvoviruses belonging to two different genera were also characterized.

**Conclusion:**

The virome in plasma samples of cattle from the forested region of Northeast China was revealed, which further characterized the diversity of viruses in cattle plasma.

**Electronic supplementary material:**

The online version of this article (10.1186/s12985-018-0923-9) contains supplementary material, which is available to authorized users.

## Background

Although large-scale cattle farms account for an increasing part in the beef industry in China, free-range cattle raising is still used especially in the Northeast China area. Because free-range cattle have close contact with farmers, virus transmission from cattle to farmers through biting flies widely distributed in forested regions of Northeast China may conceivably occur. Using a metagenomics approach we characterized viral sequences present in pools of bovine serum samples collected from a forested region of Northeast China. Small circular, Rep-encoding, ssDNA (CRESS-DNA) genomes consist of a large and highly diverse collection of viruses [1–3] that infect a wide range of cellular hosts including vertebrates (*Circoviridae*), plants (*Geminiviridae* and *Nanoviridae*), crustaceans [4, 5], and fungi (SsHADV) [6]. CRESS-DNA genomes have also frequently been detected in complex environmental samples including aquatic settings [7], insects [8–10], and animal stools [11–16]. CRESS-DNA genomes typically encode a replication initiator protein (Rep) and a capsid protein (Cap) [17, 18]. The cellular hosts of most CRESS-DNA viruses characterized in fecal or environmental samples remain elusive. Here, we report on the discovery of CRESS-DNA genomes in the blood virome of cattle raised in the forest area of Northeast China.

## Methods

### Samples and viral metagenomic analysis

During 2016, 80 bovine blood samples were collected from a forested region in Northeast China. All blood samples were taken from the caudal vein of the animals using disposable syringes and immediately transported to the laboratory on dry ice. These samples were collected as part of a survey for arthropod-borne viral infection in cattle. Library preparation and computational analysis were performed as previously described [3, 19]. Briefly, 80 plasma samples were pooled into five pools which included 16 plasma samples each. Supernatants were then collected after centrifugation (10 min, 13,000×g). Plasma pools were filtered through a 0.45-μm filter (Millipore) to remove eukaryotic- and bacterial cell-sized particles, and 200 μL of each pool was then subjected to a mixture of nuclease enzymes to reduce the concentration of free (non-viral encapsidated) nucleic acids [3]. Remaining total nucleic acid was then isolated using QiaAmp Mini Viral RNA kit (Qiagen) according to manufacturer’s protocol. Viral cDNA synthesis was performed using SuperScript III reverse transcriptase (Invitrogen) according to the manufacture’s instructions. The second strand of cDNA synthesis was performed by incubation of reverse transcribed (RT) products with Klenow Fragment DNA polymerase (New England Biolabs). Five libraries were then constructed using Nextera XT DNA Sample Preparation Kit (Illumina) and sequenced using the MiSeqIllumina platform with 250 bases paired ends with dual barcoding for each pool. Bioinformatics analysis was performed according to a previous study [20, 21]. In bioinformatics analysis, an in-house analysis pipeline running on a 32-nodes Linux cluster was developed to process the data. Cattle host reads and bacterial reads were subtracted by mapping the reads to the reference genome *Bos taurus* (assembly Bos_taurus_UMD_3.1.1) and bacterial RefSeq genomes release 66 using bowtie2 [22]. The reads whose 5 bp to 55 bp from 5 prime end is identical were considered duplicates and only one random copy of duplicates was kept. Low sequencing quality tails were trimmed using Phred quality score ten as the threshold. Adaptors were trimmed using the default parameters of VecScreen which is NCBI BLASTn with specialized parameters designed for adapter removal. The cleaned reads were *de-novo* assembled within each barcode by SOAPdenovo2 version r240 using Kmer size 63 with default settings. The assembled contigs, along with singlets were aligned to an in-house viral proteome database using BLASTx with an E-value cutoff of <10^−5^. Candidate viral hits are then compared to an in-house non-virus non-redundant (NVNR) protein database, which was compiled using non-viral protein sequences extracted from NCBI nr fasta file, to remove false positive viral hits. Contigs without significant BLASTx similarity to viral proteome database are searched against viral protein families in vFam database [23] using HMMER3 [24–26] to detect remote viral protein similarities. A web-based graphical user interface was developed to present users with the virus hits, along with taxonomy information and processing meta-information.

### Genome acquisition and PCR screening

To characterize the complete genome of the CRESS-DNA genomes, inverse PCR and Sanger sequencing was used with outward pointing PCR primers designed based on the initial Rep encoding contigs. Using the ORF finder of the Geneious software version 9, putative open reading frames (ORFs) and the stem-loop in the circular genomes were located. PCR screenings were also performed to investigate the prevalence of the five CRESS-DNA genomes in the 80 bovine plasma samples by nested PCR with specific primers designed based on the five genomes, respectively. Sequences and characteristics of the primers used in inverse and screening PCRs are shown in an additional file (see Additional file 1). PCR products were then subjected to Sanger sequencing.

### Phylogenetic analysis

For generating a phylogenetic tree, sequence alignment was performed using CLUSTAL X with the default setting [27], phylogenetic analyses were constructed through full-length Rep protein of novel CRESS-DNA viruses and other genetically-close relatives. Using the Maximum-Likelihood method, phylogenetic trees with 1000 bootstrap resamples of the alignment data sets were generated, visualized using the program MEGA [28]. Bootstrap values (based on 1000 replicates) for each node are shown if >50%.

## Result

### Overview of eukaryotic viral sequences

The 80 cattle plasma samples in the five libraries generated a total of 2,493,791 unique sequence reads using the Illumina MiSeq sequencing run with 250 bases paired ends. Sequence reads were de novo assembled using the Ensemble program [29] and compared to the GenBank non-redundant protein database using BLASTx. Results indicated that CRESS-DNA sequences accounted for a major part of total putative mammalian virus reads, with 1848 reads showing sequence similarity to the viruses from *Circoviridae* family, and 2212 reads related to viruses from *Genomoviridae* family [30]. Table 1 list the other mammalian virus sequences detected namely bovine parvovirus (BPV) (2695 reads), papillomavirus (4 reads), and picobirnavirus (3 reads), where the sequence reads of papillomavirus and picobirnavirus were shown in an additional file (see Additional file 2). Besides these mammalian viruses, a small number of insect viral sequences were detected showing low-level sequence similarity to *Iflaviridae* and *Dicistroviridae*. The CRESS-DNA viruses showed lower levels similarities to known genomes available in GenBank, whose divergent genomes were then further characterized.

**Table 1:** Characterization of the viral sequence reads in cattle blood samples

Library ID	Total unique reads	Percentage of host reads	Family	Genus	Species	GenBank no. of the matches	Aa identities with the match(s)	Total reads
Cattle01	174,181	55%/10%	*Parvoviridae*	Erythroparvovirus	BPV3	AF406967	98–100%	809
Cattle02	642,907	53%/12%	*Parvoviridae*	Erythroparvovirus	BPV3	AF406967	98%	205
			*Papillomaviridae*	Xipapillomavirus	Bovine papillomavirus	AB543507 KM983393 DQ098911	81%–95%	4
			*Picobirnaviridae*	Picobirnavirus	Dromedary picobirnavirus	KM573798	80%–84%	3
Cattle03	458,532	49%/21%	*Parvoviridae*	Copiparvovirus	BPV2	KT148961	86%	353
			*Parvoviridae*	Erythroparvovirus	BPV3	AF406967	95%–97%	477
Cattle04	154,826	67%/9%	*Parvoviridae*	Erythroparvovirus	BPV3	AF406967	94%–97%	432
Cattle05	1,063,345	42%/19%	*Parvoviridae*	Copiparvovirus	BPV2	KT148961	94%	46
			*Parvoviridae*	Erythroparvovirus	BPV3	AF406967	93%	373

*BPV* Bovine parvovirus

### CRESS-DNA genomes

**Fig. 1:**
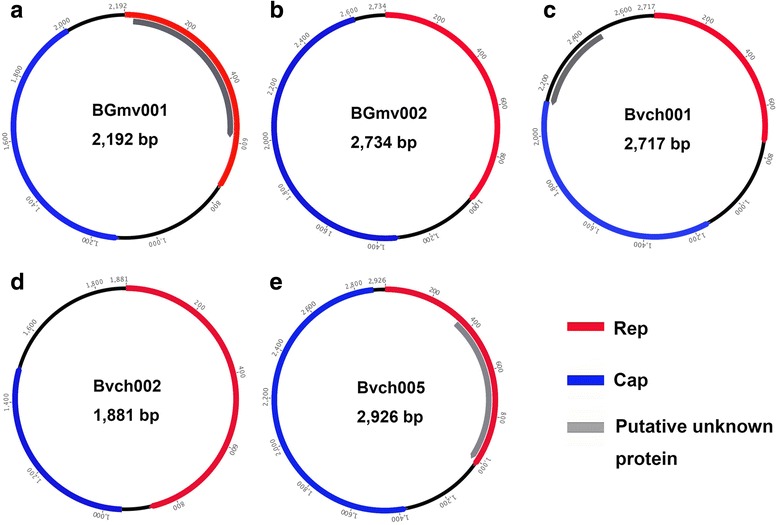
The genomic organization of the five CRESS-DNA viruses identified in the cattle blood samples

Two complete CRESS-DNA genomes showing the highest sequence similarity to viruses from *Genomoviridae* family were obtained through inverse PCR based on two large contigs from these libraries and Sanger sequencing. Genomes were 2192 nt (BGmv001, from library 5) and 2926 nt (BGmv002, from library 1) in length, respectively. As shown in Fig. 1a and b, the two genomes contained two bidirectional ORFs, encoding the putative Rep and Cap proteins. The two gemycircularviruses (the only genus currently in the *Genomoviridae* family) in this study did not contain putative intron in the Rep gene. BLASTp search in GenBank based on the amino acid sequence of Rep showed BGmv001 shared the highest sequence similarity of 56% to a gemycircularvirus genome sequenced from dragonfly abdomens (NC_023872) [8]. BGmv002 shared the highest protein sequence similarity of 36% to a gemycircularvirus from bird feces (KF371630) [11]. Figure 1 C showed the CRESS-DNA genome of Bvch001 with 2717 nt in length, whose Rep and Cap are arranged in inverse direction. The best matches in GenBank of Bvch001 based on amino acid sequence of Rep include two smacoviruses from Gorilla (KP233191 and KP233192) [31], two unidentified circular ssDNA virus from Rhesus macaque (KU043429 and KU043430) [32] and four porcine stool-associated circular viruses (KJ577812-KJ577815) [33], sharing 52% sequence similarity with them. Two complete CRESS-DNA genomes showing the highest similarity to circoviruses were determined through inverse PCR based on two large contigs from these libraries and Sanger sequencing, which were 1881 nt (Bvch002, from library 5) and 2926 nt (Bvch005, from library 4) in length, respectively. Figure 1 D and E indicated the genomic organization of Bvch002 and Bvch005, where the predicted Rep and Cap of Bvch002 are arranged in the same direction within the genome and those of Bvch005 are inversely arranged. BLASTp search in GenBank based on the amino acid sequence of Rep showed Bvch002 shared the highest sequence similarity of 38% to a circovirus-like virus from dragonfly (KM598397) and BGmv005 shared the highest sequence similarity of 48% to a bovine faeces-associated circular DNA virus (NC_030137) [34].

**Fig. 2:**
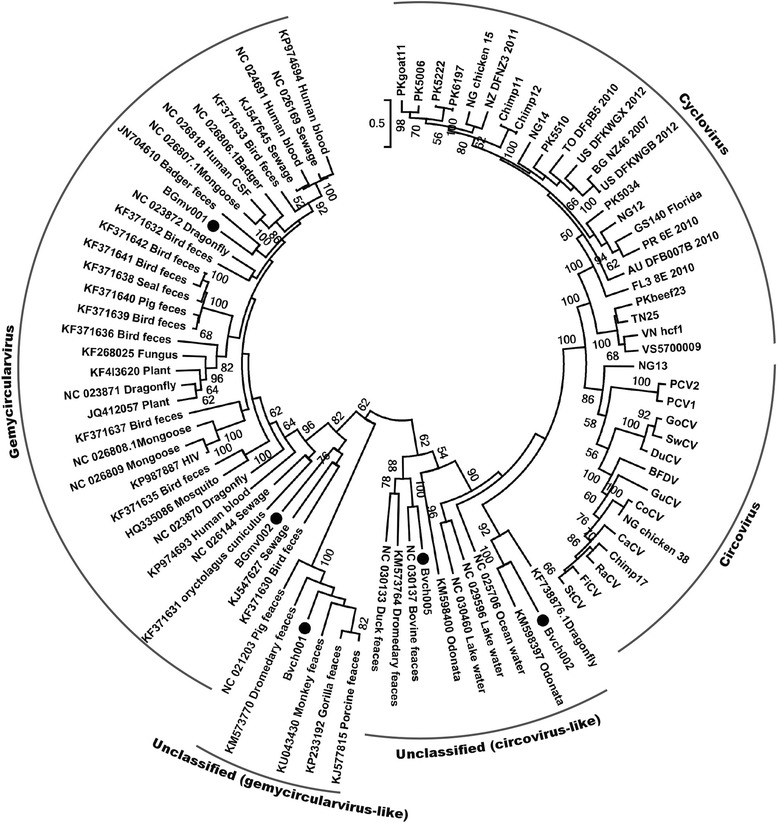
Phylogenetic analysis of the five CRESS-DNA viruses identified in the cattle blood samples**.** Phylogenetic analysis was performed based on the amino acid sequence of Rep protein. The sequence alignments included five CRESS-DNA viruses identified in the cattle blood samples in this study, their best BLASTp matches in GenBank based on the Rep proteins, and the representative strains of circovirus, cyclovirus, and gemycircularvirus. Host or sources of the closely related viruses of the five CRESS-DNA viruses in this study in the phylogenetic analysis were showed on branches. Viruses identified in this study were labeled with black dots

Based on the alignment of the Rep amino acid sequences herein detected with the best matches of BLASTp search in GenBank and those of representative CRESS-DNA genomes including circovirus, cyclovirus and gemycircularvirus, a phylogenetic tree was constructed. As shown in the Fig. 2 BGmv001 and BGmv002 belong to the cluster of gemycircularvirus, Bvch002 and Bvch005 fall into the clade showing close relationship with circovirus, and Bvch001 clustered into the group neighbouring to the gemycircularvirus clade.

PCR screening results indicated Bvch001 was detected in three samples, and sequencing results indicted the three 310 bp sequence fragments were different from each other, sharing >99.3% similarity (the two new sequences were deposited in GenBank with nos. MG601761 and MG601762). BGmv001 was detected in two samples and one of them (with GenBank no. of MG601763) had one base pair difference from the original genome based on the 290 bp PCR fragment. Two samples were positive for BGmv002 and the two sequences had two base pairs difference based on the 280 bp PCR product (the GenBank no. of new sequence is MG601764). The rest two CRESS-DNA genomes, Bvch002 and Bvch005, were respectively detected in a single sample, where their sequencing results were identical to the original genomic sequences, respectively.

### Bovine parvovirus

**Fig. 3:**
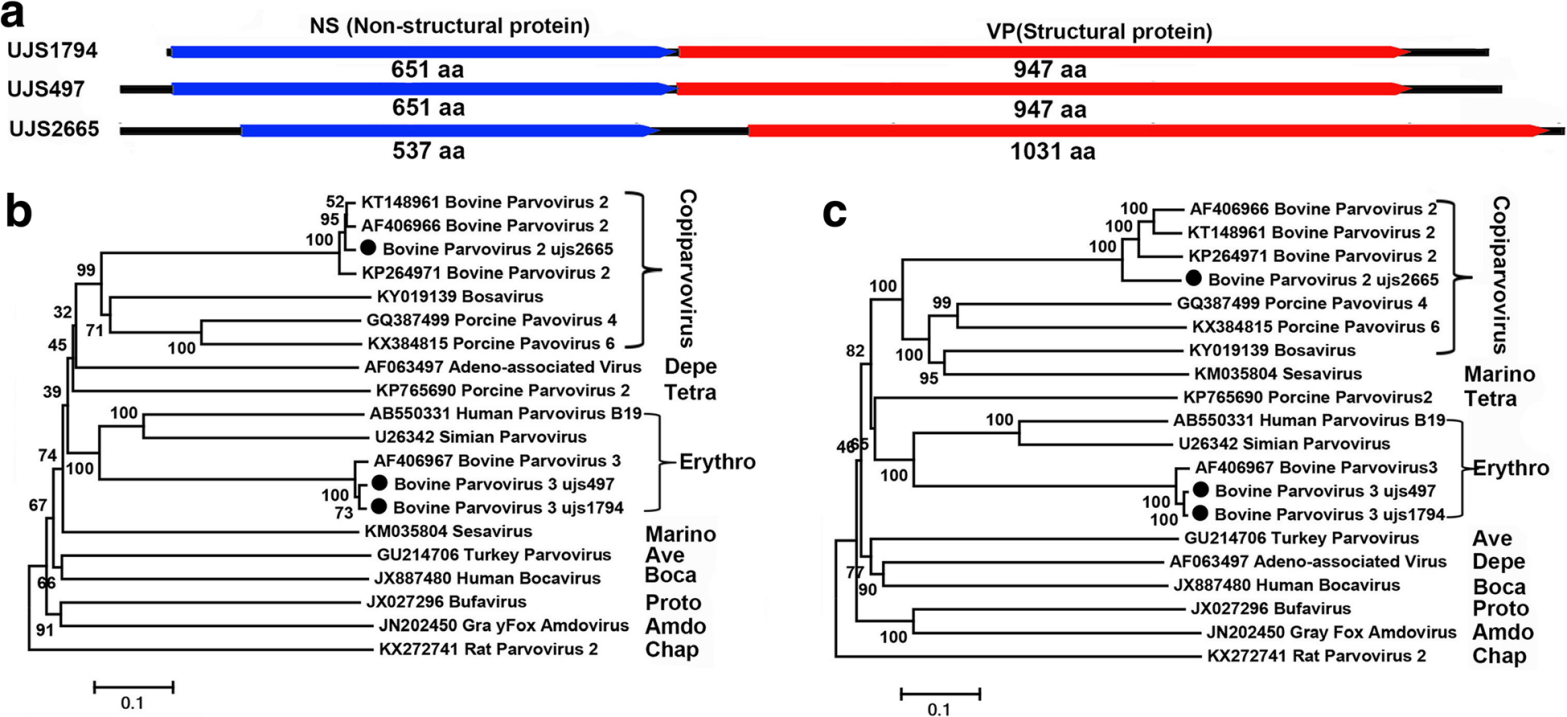
The genomic organization of the three BPVs and phylogenetic trees based on the nonstructural (NS) and VP1 proteins, respectively. **a** The genomic organization of the three BPVs identified in the cattle blood samples. **b** and (**c**) Phylogenetic analysis based on the amino acid sequence of NS and VP proteins, respectively. The sequence alignments included three BPVs identified in the cattle blood samples in this study, their best BLASTp matches in GenBank, and the representative members of the related genera in family *Parvoviridae*. Viruses identified in this study were labeled with black dots

Bovine parvovirus 3 (BPV3) was detected in all the five cattle plasma pools and BPV2 was detected in two pools. Three complete coding regions of BPV genomes could be assembled, where one (with stain name ujs2665) belongs to BPV2 (from CattleB03) and two (with stain name ujs1794 and ujs497) belong to BPV3 (from CattleB03 and CattleB04, respectively). Figure 3 showed the genomic organization of the three BPVs and phylogenetic trees based on the nonstructural (NS) and VP1 proteins, respectively. The BPV2’s NS showed 96–98% identities to the 3 BPV2 genomes currently available in GenBank database, while the VP protein showed 84–86% identities to them, suggesting that ujs2665 is a putative new strain in BPV2 group. The NS proteins of the two BPV3 shared 98% sequence identity with each other and both showed 96% sequence identity with the only BPV3 strains in GenBank, and the VP proteins of them shared 99% sequence identity with each other and 97% identity to the BPV strain.

### Nucleotide sequence accession numbers

The viral genomes described in detail here and the PCR screening sequence fragments were deposited in GenBank under the following accession numbers MG026727- MG026729, MF669476- MF669480, and MG601761- MG601764. The raw sequence reads from the viral metagenomic libraries were deposited in the Short Read Archive of GenBank database under the accession number SRX3235902.

## Discussion

Our analysis characterizing enriched viral particle associated nucleic acids in cattle plasma showed that CRESS-DNA virus and bovine parvovirus sequences are present in all the five libraries and yielded the most reads relative to other viruses. In the recent years, a large number of CRESS-DNA genomes have been determined in mammals, birds, insects, plants, fungi, and environment samples which bringing to light a high level of genetic diversity among these virus [1–16]. In this study, we detected three different groups of CRESS-DNA genomes in the plasma of free-range cattle in north-east China, which belonged to gemycircularvirus and two unclassified groups, respectively, based on phylogenetic analysis. Although detecting CRESS-DNA genomes in blood, a site considered to be sterile, makes it reasonable to believe the possibility of these viruses’ replication in the cell of cattle host, reports confirming replication in mammalian cells or of sero-conversion to these viruses are still lacking. It is also conceivably that CRESS-DNA genomes in blood results from skin contamination during phlebotomy, translocation from ingested food products or enteric organisms such as protozoa or fungi, or reflect systemic bovine infectious with still unknown cellular hosts with release of their viruses into the blood stream.

Bovine parvoviruses include BPV1, 2 and 3 and the recently described bosavirus belonging to three different genera of the *Parvoviridae* family. BPV most commonly causes diarrhoea in neonatal calves and respiratory and reproductive disease in adult cattle [35, 36]. In the present study, BPV3 was detected in all the five cattle plasma pools and BPV2 was detected in two pools, showing high viral sequence reads percentages. These cattle included in the present were all in normal status without clinical symptoms, thus detecting BPV3 with high rate in plasma samples from healthy cattle suggested this virus were prevalent in high rate in adult cattle in this area. BPV2 has also been reported in serum from calves in the US [37, 38].

The other four types of virus including papillomavirus, picobirnavirus, pegivirus, and bovine torovirus were also detected in cattle plasma pools with low percentage of sequence reads. The papillomavirus sequences detected in pool ID cattle02 were closely related to viruses detected in bovine teat papillomas [39]. The other pool ID cattle03 contained papillomavirus sequences showing <50% sequence similarity to human papillomavirus, which may represent a novel species of papillomavirus. Whether these papillomavirus sequences reflect contamination with human skin or bovine papillomaviruses possibly introduced into the serum pools from cattle skin during sampling is unknown. Picobirnavirus are widely present in animal fecal samples [40, 41], although they were detected in one plasma pool in the present study, they might be from the contamination of feces which is very common on bovine tail skin. Whether these viruses detecting in cattle plasma can cause disease needs further research.

In the investigation of virome, most research groups performed viral purification by filtering samples through small-pore (0.2–0.45 μm) filters, which prevented the detection of viruses larger than the filter pores, e.g. the members of the order Megavirales [42]. In our studies, considering that most members of giant viruses have diameter < 0.45 μm, we only used the 0.45 μm filters, which did not have significant effect on the virome results [21, 43].

## Conclusion

The virome in plasma samples of cattle from the forested region of Northeast China included the viruses belonging to the families including *Parvoviridae, Papillomaviridae, Picobirnaviridae,* and viruses showing sequence similarity to CRESS DNA viruses, where 5 divergent genomes of CRESS-DNA viruses and 3 genomes of bovine parvoviruses were characterized in detail. This study increased the knowledge of the diversity of viruses in cattle plasma.

## Additional files

Additional file 1: Primers used in the specific screening PCR and inverse PCR. (PDF 54 kb)

Additional file 2: The sequence reads of papillomavirus and picobirnavirus in library cattle02. (PDF 33 kb)

## Abbreviations

BLAST: Basic Local Alignment Search Tool
BPV: Bovine parvovirus
CRESS: Circular Rep-encoding single stranded
ORF: Open reading frame
PCR: Polymerase chain reaction
