# Supplementary material for: Plasma virome of cattle from forest region revealed diverse small circular ssDNA viral genomes
Source: Virol J. 2018 Jan 15;15:11. doi: 10.1186/s12985-018-0923-9 (PMC5769433; doi:10.1186/s12985-018-0923-9)
Supplement: Supplementary file 1 — Primers used in the specific screening PCR and inverse PCR. (PDF 54 kb) [file 12985_2018_923_MOESM1_ESM.pdf]

Additional file 1: Primers used in the specific screening PCR and inverse PCR.

Primer’s ID	Target virus	Application	Sequence(5’-3’)	Fragment size(bp)	Annealing temperature(°C)	No. of cycles
BC-CH1 WS	Bvch001	Screening (first round)	AGAGGGGGTCAGCAAGAAGA	560	50	30
BC-CH1 WX			CACGGGACAACTTGCCTAGT			
BC-CH1 NS	Bvch001	Screening (second round)	GCATCGGACACATGGGACTA	350	53	32
BC-CH1 NX			TGATGAGCCCGTCCTTTATGG			
BC-CH2 WS	Bvch002	Screening (first round)	TCTTGACCACTAAGCCGTATGT	580	48	30
BC-CH2 WX			GACTACAGCACTGGAGCTCTT			
BC-CH2 NS	Bvch002	Screening (second round)	AGAGGAACAACACAACAGAACAT	330	53	32
BC-CH2 NX			GCCTTTATAACCATCCCACCATTT			
BC-CH5 WS	Bvch005	Screening (first round)	TGGTTCTTGGGTGGTATAGTTTC	650	49	32
BC-CH5 WX			AGACTCTCACTTACACGCTTTCA			
BC-CH5 NS	Bvch005	Screening (second round)	AGTGGAAGGTGCCTGGTCTA	320	53	32

BC-CH5 NX			AACTTCTGCACACACGGTAT			
BG-CH1 WS	BGmv001	Screening (first round)	CCCCAATGATCGTGCCCATA	640	48	32
BG-CH1 WX			CATGTTGATGGCGGAACTCAT			
BG-CH1 NS	BGmv001	Screening (second round)	AAGCGACGGGTAAGATAACC	330	53	32
BG-CH1 NX			CATCATACGGCACACCTGAAA			
BG-CH2 WS	BGmv002	Screening (first round)	TGAACTCTTTCTGAGCTCCAAAGA	550	48	32
BG-CH2 WX			AGCTCGTGGTTTGACTAAGG			
BG-CH2 NS	BGmv002	Screening (second round)	TCTGGCCCATTCAGTCTTACC	320	53	32
BG-CH2 NX			ACTGGCATCCTTCCACTGAAA			
R-NS002C	Bvch002	Inverse PCR	TCTCAGGCTGCATCCACACAT	620	54	32
R-NX002C			ACTACGTGCGGTAGAGCTGG			
R-NS005C	Bvch005	Inverse PCR	TCCCAGTGCCAGTACACATAC	450	53	32
R-NX005C			CTCCGAGGGTGCAAGAAGTT			
R-NS001G	BGmv001	Inverse PCR	CCACAACACGAGGGATAATTTTC	350	56	32

R-NX001G			GGGGAGACACGCCTATTTTG			
R-NS002G	BGmv002	Inverse PCR	TCACCGTGTCACCCATACTC	580	56	32
R-NX002G			GGCATTCTTTGGAGCTCAGAAA			
