# Supplementary material for: Plasma virome of cattle from forest region revealed diverse small circular ssDNA viral genomes
Source: Virol J. 2018 Jan 15;15:11. doi: 10.1186/s12985-018-0923-9 (PMC5769433; doi:10.1186/s12985-018-0923-9)
Supplement: Supplementary file 2 — The sequence reads of papillomavirus and picobirnavirus in library cattle02. (PDF 33 kb) [file 12985_2018_923_MOESM2_ESM.pdf]

>CattleB02Bovine_Papillomavirus
TTTGACAGCCCTCATCAAACTCATTACAAACATAGATTTGCCTGTATTAGG
TGGGCCTACAATCACCATGCAGTTCTTCTTAGGAACACTGTGCAACAAAT
CTTTGAAAGCAGCTAGGAAACTTAAAAAATTCACATTTTGAAACTTTAGA
AACTGCACAATTGTTTTAAAGTCTCCCGTCCCCTCTATTTGACTAATACAT
TTCTTGATCCACTGCCCCATAGTCATTTCTCTCATTTCAGCGG
>CattleB02Bovine_papillomavirus
ATCAGTCCAGTTAGTTTAAATTGTGAAGAAGAGGAATTAGAGGTTGAGGA
AGTAGATTGCCCTAATCCTTATGCAATTGAGACAGCTTGTTATGTTTGTGA
AGACATACTACGCATAGCTGTTGTAACCTCGAACGACGGAATCCGGGACC
TGCAACAACTGCTGCTGAGCAGCCTTTTTCTACTGTGTGCAAGTTGTTCTA
GAGAAGCTTTTTGCCTTCGCAGAGCGCATCAGAATGGATAAAGGTATT
>CattleB02Bovine_Papillomavirus
ATTGCATATTATTATGCTAGGTTAGCTGAGGTGGATTCTAATGCTGCAGCC
TTTTTGAAGTGCAACAACCAGGTTAAGCATGTGAAAGAGTGTGCTCAAAT
GACTAGGTACTACAAAACCGCTGAAATGAGAGAAATGACTATGGGGCAG
TGGATCAAGAAATGTATTAGTCAAATAGAGGGGACGGGAGACTTTAAAAC
AATTGTGCAGTTTCTAAAGTTTCAAAATGTGAATTTTTTAAGTTTCCTAG
>CattleB02Bovine_Papillomavirus
GTTACTCTGACTGGTTATGCATTGTAAAGGCTTACGAGGTGGCAGTGTTAA
CTTTCGTTTTGCAGCGCTGATCTCCGTATGGAAATCGAAAGCCTCCTGTTG
TGCAAACAATTCTGCGGAATTCCCTTGCTCGCATTCAGCATTATCAATTAG
GTCAGACAAATCAGACACACTTTCTTCACACCCAACTTCACTATCAGATTG
ACTACATTGTGCTTGATCGTCTA
>CattleB02Dromedary_picobirnavirus
ATTATGCCTAAAAATAATGAAACTAAATGGAAGGAAATTCAATTTGATGA
TTGTTTTAACCTTCCCAATCCAGGTTTACGGTCTTATTTTGACATAGTCAGA
AAAGGACAACCTGACGAGTACAGAACCACCTTTGCAAAGGGTGACTCGTT
AAATAAAGTTCTGAACGATTGGAGACCTACACTTGAGTCGTTATCTGACA
AGTGGCCTACACTTGTAGATTTTGAAAACGACTTAAAGGCTAAGGTCG
>CattleB02Dromedary_picobirnavirus
CTTTAATCTGCCTAATCCAGGGCTTCGGTCTTATTTTGACATCGTCAGAAA
AGGCCAACCCGACGAGTACAGGACCACTTTCGCCAAGGGTGATTCGGTCG
AGAAGGTCCTGAACGATTGGAGTTCCACACTCGAATCGGTCGC
>CattleB02Dromedary_picobirnavirus
TAATGATCGATGTCTTCAAGCCTTTCAGACAGTGGCTTCATGATAGACAGC
GGTCCGACCTTAGCCTTTAAGTCGTTTTCAAAATCTACAAGTGTAGGCCAC
TTGTCAGATAACGACTCAAGTGTAGGTCTCCAATCGTTCAGAACTTTATTT
AACGAGTCACCCTTTGCAAAGGTGGTTCTGTACTCGTCAGGTTGTCCTTTT
CTGACTA
